# *Nav2 *is necessary for cranial nerve development and blood pressure regulation

**DOI:** 10.1186/1749-8104-5-6

**Published:** 2010-02-25

**Authors:** Elizabeth M McNeill, Kenneth P Roos, Dieder Moechars, Margaret Clagett-Dame

**Affiliations:** 1 Interdepartmental Graduate Program in Nutritional Sciences, University of Wisconsin, Madison, WI, USA; 2 Department of Physiology and the Cardiovascular Research Lab, David Geffen School of Medicine at UCLA, Los Angeles, CA, USA; 3 Johnson & Johnson Pharmaceutical Research and Development, Beerse, Belgium; 4 Department of Biochemistry, University of Wisconsin, Madison, WI, USA; 5 Pharmaceutical Science Division, University of Wisconsin, Madison, WI, USA

## Abstract

**Background:**

All-*trans *retinoic acid (atRA) is required for nervous system development, including the developing hindbrain region. Neuron navigator 2 (*Nav2*) was first identified as an atRA-responsive gene in human neuroblastoma cells (retinoic acid-induced in neuroblastoma 1, *Rainb1*), and is required for atRA-mediated neurite outgrowth. In this paper, we explore the importance of *Nav2 *in nervous system development and function *in vivo*.

**Results:**

*Nav2 *hypomorphic homozygous mutants show decreased survival starting at birth. *Nav2 *mutant embryos show an overall reduction in nerve fiber density, as well as specific defects in cranial nerves IX (glossopharyngeal) and X (vagus). *Nav2 *hypomorphic mutant adult mice also display a blunted baroreceptor response compared to wild-type controls.

**Conclusions:**

*Nav2 *functions in mammalian nervous system development, and is required for normal cranial nerve development and blood pressure regulation in the adult.

## Background

The vitamin A (retinol) metabolite all-*trans *retinoic acid (atRA) is essential for normal development of the vertebrate nervous system. During early development, atRA plays a role in patterning the hindbrain and in neuronal specification [1-5]. At later stages of development, atRA is needed for neuronal elongation and axonal pathfinding [6,7]. Vitamin A deficiency has been shown to alter neurite outgrowth from the spinal cord and hindbrain regions in the developing chick, rat and mouse [8-10]. In vitamin A deficient rat embryos, hindbrain patterning is rescued by a level of atRA that is still inadequate to support normal development of the most posterior cranial nerves [9]. In culture, atRA has been shown to increase neurite outgrowth from embryonic sympathetic and dorsal root ganglia neurons and explants [11-15], embryonic spinal cord explants [12,16], and neuroblastoma (NB) cell lines [17,18]. However, the mechanism whereby atRA acts to produce these cytoskeletal changes is largely unknown.

The level of atRA in the central and peripheral nervous system of vertebrates [19-21] is regulated through differential expression of both synthetic (*Raldh*) [22-24] and catabolic enzymes (*Cyp 26 *family) [5,25]. atRA binds to nuclear retinoic acid receptors (*Rarα*, *Rarβ*, *and Rarγ*) that together with the retinoid X receptor regulate the expression of atRA target genes [26]. atRA has been shown to regulate the expression of 3' homeobox genes, which are essential for normal hindbrain patterning. However, genes that lie downstream of atRA and its receptors that are involved in producing changes in neurite outgrowth and axonal elongation remain to be elucidated.

Using a human NB cell line (SH-SY5Y) that extends neurites in response to atRA, our group identified the atRA-responsive gene, retinoic acid-induced in neuroblastoma 1 (*Rainb1*) [27], which was renamed neuron navigator 2 (*Nav2*) [28]. *Nav2 *has also been identified by others as *Pomfil2 *(pore membrane and/or filament interacting-like protein) [29] and *Helad1 *(helicase, APC-downregulated) [30]. *Nav2 *is rapidly induced (within 4 hours) by atRA and has been detected in the developing rat nervous system, where its expression is sensitive to both high and low levels of atRA [27]. Loss-of-function studies show that *Nav2 *induction is required for atRA to induce neurite outgrowth in human NB cells [31].

*Nav2 *is a member of the neuron navigator family comprising *Nav1*, *2 *and *3 *[28]. The *Nav2 *gene is composed of 38 exons, and the largest open reading frame encodes a protein of 261 kDa. Several alternatively spliced variants have been identified, and a shorter protein based on an alternative start site upstream of exon 13 has been proposed based on PCR studies [32]. Of the three *Nav *family members, *Nav2 *shows most similarity to the *Caenorhabditis elegans *homolog *unc-53*, which is essential in the longitudinal migration of several cell types, including neurons, developing sex myoblasts, and the excretory cell [33-36]. In the nervous system, *unc-53 *is required for normal mechanosensory neuron elongation [36,37]. Transgene expression of human full-length *Nav2 *rescues the defects in *unc-53 *mutant mechanosensory elongation [6,31]. Thus, studies both in *C. elegans *as well as in cultured human NB cells support a role for *Nav2 *in neurite outgrowth and axonal elongation.

The acuity of several sensory systems (olfactory, auditory, visual) and the ability to sense pain is impaired in the adult hypomorphic *Nav2*/*unc-53H2 *mutant mouse [32]. The *unc-53H2 *mutant was generated using a gene trap method in which insertion of a neo cassette occurred between exons 7 and 8 of the *unc-53H2 *(*Nav2*) gene, abolishing expression of the full-length *Nav2 *transcript and protein, but leaving expression of the shorter transcript undisturbed. The long transcript is required for atRA to induce neurite outgrowth in human NB cells [31] and is expressed most abundantly in the nervous system [27]. In the present work, we examine development of the embryonic nervous system in the *Nav2*/*unc-53H2 *mutant, with particular emphasis on the hindbrain region, known to be particularly sensitive to the adverse effects of vitamin A deficiency. In addition, the function of hindbrain nerves in the maintenance of blood pressure in the adult *Nav2*/*unc-53H2 *mutant is examined.

## Results

### Postnatal survival is reduced in *Nav2 *hypomorphic mutant mice

**Figure 1:**
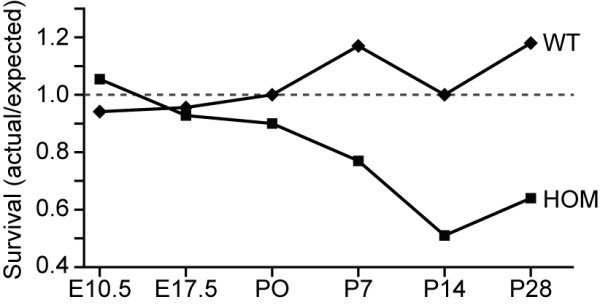
**Postnatal survival is reduced in *Nav2*^-/- ^mice**. Female *Nav2 *heterozygotes were mated with *Nav2*^+/- ^and *Nav2*^-/- ^males, and litters were examined and genotyped at embryonic days (E) 10.5 and 17.5, and postnatal days (P) 0, 7, 14, and 28; with a total of 90, 188, 348, 94, 93, and 791 offspring examined at each time point, respectively. A reduction in expected number live animals (<1.0) appears in the homozygote mutant (HOM) group after P0. At weaning (P28), only 60% of the expected number of homozygous mice are alive. Wild-type (WT) values remain around the expected ratio of 1.0 (indicated by the dotted line) at all time points.

When examined at embryonic day (E) 10.5 or E17.5, no significant reduction in the expected number of homozygous mutant offspring was observed; however, increased lethality was observed after postnatal day (P)0 (Figure 1). At P0, the number of homozygous *Nav2 *hypomorphic mice was 10% lower than expected, and by P28, this number had risen to 36%. The reason for early neonatal death was sought by examining *Nav2*^-/- ^mice that died shortly after birth (P0). By external examination, there was never any evidence of a milk spot in the stomach, indicating that the neonates failed to suckle. In addition, when the *Nav2*^-/- ^neonates (n = 3) were fixed and subjected to serial sectioning followed by hematoxylin and eosin staining, none of the mice showed evidence that the lungs had ever inflated (data not shown). This is consistent with the finding of a persistent opening of the foramen ovale, an opening between the right and left atrium that normally closes at birth after the lungs fill with air and the pulmonary capillary bed opens (data not shown). The cause of neonatal death at later times was not determined, although it did not appear to be related to an inability to suckle, as the weight of *Nav2*^-/- ^neonates at P7 and P14 did not differ from that of the wild-type controls (data not shown).

### Overall nerve density is reduced in the *Nav2 *hypomorphic mutant

**Figure 2:**
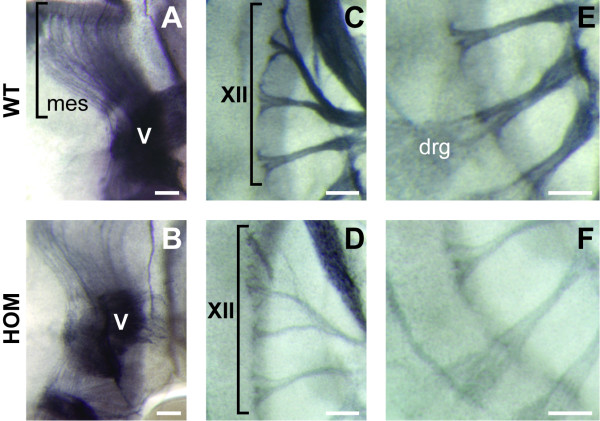
**Nerves are less densely stained by an antibody to neurofilament in *Nav2 *homozygous mutant embryos at E11.5**. The **(A) **mesencephalic tract (mes) nerve fibers, **(C) **cranial nerve XII, and **(E) **dorsal root ganglia (drg) nerves are shown in wild-type (WT) mice. **(B, D, F) ***Nav2*^-/- ^littermates show decreased staining of nerve fibers in these same regions. The images shown here are representative of the majority of those scored in Table 1 as positive for a reduction of nerve fiber density. Scale bar: 100 μm.

In order to determine whether the atRA-responsive gene *Nav2 *influences early nervous system development, hypomorphic *Nav2*^-/- ^mutant embryos were stained with an antibody to neurofilament protein (2H3) and compared to wild-type controls at E11.5. The expression of a reporter gene driven by *Brn3a *in the developing sensory nerves was also examined at E12.5, E13.5, and E15.5 of development. An overall decrease in neurofilament staining was particularly notable in the cranial nerves as well as the mesencephalic tract and the dorsal root ganglion (DRG) neurons in *Nav2*^-/- ^hypomorphic mutant mice relative to their wild-type littermates (Figure 2). To quantify the overall decrease in nerve fiber density in these regions, wild-type and homozygous embryos from *Nav2*^+/- ^× *Nav2*^+/- ^crosses were scored blinded for relative density of neurofilament staining across litters from the same staining runs. Nearly 40% of the *Nav2*^-/- ^embryos were classified as exhibiting decreased nerve fiber density, while only 7% of the wild-type animals scored positive for this effect (Table 1). The percentage of affected heterozygote embryos was intermediary between that of the homozygotes and wild-type controls (data not shown). The number of homozygous mutants showing a reduction in nerve fiber density was found to be statistically different from their wild-type counterparts (Table 1).

**Table 1:** Percentage of animals with decreased nerve fiber density

Genotype	Decreased nerve fiber density
Wild type	7% (2/27)
Homozygote	39% (37/95)

Values shown in parentheses are the number of affected embryos over the total number of embryos evaluated; Z-score = -3.1; *P*-value ≤ 0.01. The determination of a decrease in nerve fiber density was made when an embryo showed an overall reduction in neurofilament staining in the region of the cranial nerves (V to XII) compared to wild type littermates. In addition, a subset of embryos (23 wild-type and 63 *Nav2*^-/-^) was also scored for reduction in staining in the mesencephalic tract and dorsal root ganglion neurons. A similar percentage of homozygote embryos showed a reduction of nerve fiber density in both regions (30%), whereas only 4% of the wild-type group was affected.

**Figure 3:**
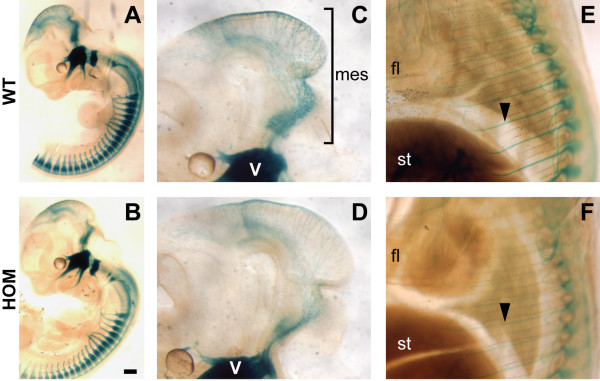
***Nav2*^-/-^/*Brn3a-lacZ*^+/- ^mice show a reduction in *lacZ *staining of sensory nerves compared to their *Nav2*^+/+^/*Brn3a-lacZ*^+/- ^littermates**. **(A) ***Nav2 *wild-type (WT) and **(B) **homozygote mutant (HOM) littermates in a *Brn3a-lacZ*^+/- ^background shown at the same developmental stage (E13.5). **(C, D) **Specifically, there appear to be fewer sensory neurons in the mesencephalic tract (mes) region in the *Nav2*^-/-^/*Brn3a-lacZ*^+/- ^embryo (D) (E13.5) compared to the wild-type (WT) littermate (C) (E13.5). **(E, F) **A reduction in spinal nerve staining is also observed in the *Nav2*^-/-^/*Brn3a-lacZ*^+/- ^embryo at E15.5 (compare (F) to the wild-type in (E)). The results shown are representative of nine wild-type and nine *Nav2*^*-/- *^embryos examined at E12.5 to 13.5, and two wild-type and three *Nav2*^*-/- *^embryos at E15.5. Scale bar: 200 μm. fl, forelimb; st, stomach.

The development of the sensory neurons was further examined by crossing a *Brn3a-lacZ *reporter mouse strain [38] into the *Nav2 *mutant background. *Brn3a *is a POU-domain transcription factor expressed in primary sensory neurons of the cranial and dorsal root ganglia and in specific neurons in the caudal central nervous system. *Brn3a-lacZ *heterozygotes appear to show normal sensory neuron development [39]. Using this reporter, an overall reduction in sensory nerve fiber staining was observed in the mesencephalic tract, at E12.5 and E13.5 (Figure 3 and data not shown), and in the spinal nerves at E15.5 in the homozygote mutants compared to wild-type and heterozygous controls (Figure 3).

### *Nav2 *hypomorphic mutants have defects in cranial nerves IX and X

**Figure 4:**
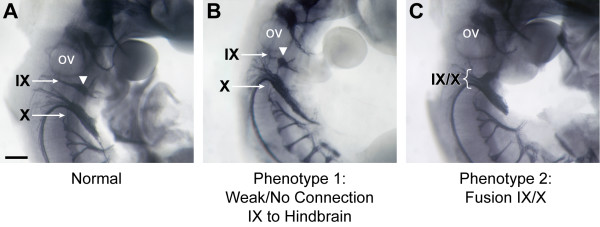
**Cranial nerve IX and X defects are found in homozygous *Nav2 *hypomorphic mutants**. Whole-mount immunostaining for neurofilament protein in **(A) **a normal *Nav2*^*+/+ *^embryo, and in **(B, C) ***Nav2*^*-/- *^embryos at E11.5. In the *Nav2*^*-/- *^embryo with phenotype 1 (B), the nerve fibers connecting the distal ganglia of IX (white arrowhead) to the proximal ganglia and hindbrain exit point are missing. In the *Nav2 *mutant showing phenotype 2 (C), the IXth and Xth nerves are fused (brackets). Cranial nerves are indicated in roman numerals: IX, glossopharyngeal; X, vagus; ov, otic vesicle. Scale bar: 200 μm.

The development of the cranial nerves (CNs) was examined by staining embryos at E11.5 in whole-mount with an antibody to neurofilament protein. While the gross organization of CN V (trigeminal), VII/VIII (vestibuloacoustical), XI and XII in the *Nav2*^-/- ^mutants did not appear different from the wild-type controls, this study revealed the development of CN IX (glossopharyngeal) alone, or together with CN X (vagus), was altered in a significant number of *Nav2*^-/- ^hypomorphic embryos. Normally at this stage of development, the distal ganglia of CN IX is connected to the proximal ganglia and the neural tube by tightly fasiculated axons, originating both from the neurons of the distal ganglia (placode-derived) as well as from axons projecting from the proximal ganglion neurons (neural crest-derived) and the motor neurons originating at the level of rhombomere (r)6/7 of the neural tube (Figure 4A). The most frequently observed phenotype in the *Nav2*^-/- ^hypomorphic embryos was either the complete loss of nerve fibers connecting the distal ganglia of CN IX to the hindbrain or else a severe reduction in the density of this nerve branch (phenotype 1; 39% or 52 out of 133 embryos; Figure 4B). In the second phenotype, the IXth and Xth nerves were fused (Figure 4C), whereas these nerves are normally well separated at this stage (Figure 4A). Phenotype 2 was observed in 38 out of 133 or 29% of homozygous Nav2 hypomorphic embryos. Overall, the penetrance of phenotypes 1 and 2 together was 60%, with nearly half of the *Nav2*^-/- ^embryos affected on both sides. There was a statistically significant difference in incidence of CN IX/X defects in the Nav2^-/- ^hypomorphs compared to the wild-type group for each phenotype (1 and 2), as well as in combination (Table 2).

**Table 2:** Percentage of embryos (sides) examined with cranial nerve abnormalities

Genotype	Phenotype 1: gIX weakly or not connected to hindbrain	Phenotype 2: CN IX/X fusion	Phenotypes 1 and 2 combined
Wild type	7% (4/59)	7% (4/59)	14% (8/59)
Homozygote	27% (72/266)	17% (45/266)	44% (117/266)

Values shown in parentheses are the number of abnormal observations over the total number of observations (60 wild-type and 133 homozygous embryos were scored on both the right and left side; one wild-type side (observation) was lost). Phenotype 1: Z-score = -3.33 and *P*-value ≤ 0.001. Phenotype 2: Z-score = -1.97 and *P*-value ≤ 0.05. Phenotypes 1 and 2 combined: Z-score = -4.35; *P*-value ≤ 0.0001. gIX, ganglia IX.

**Figure 5:**
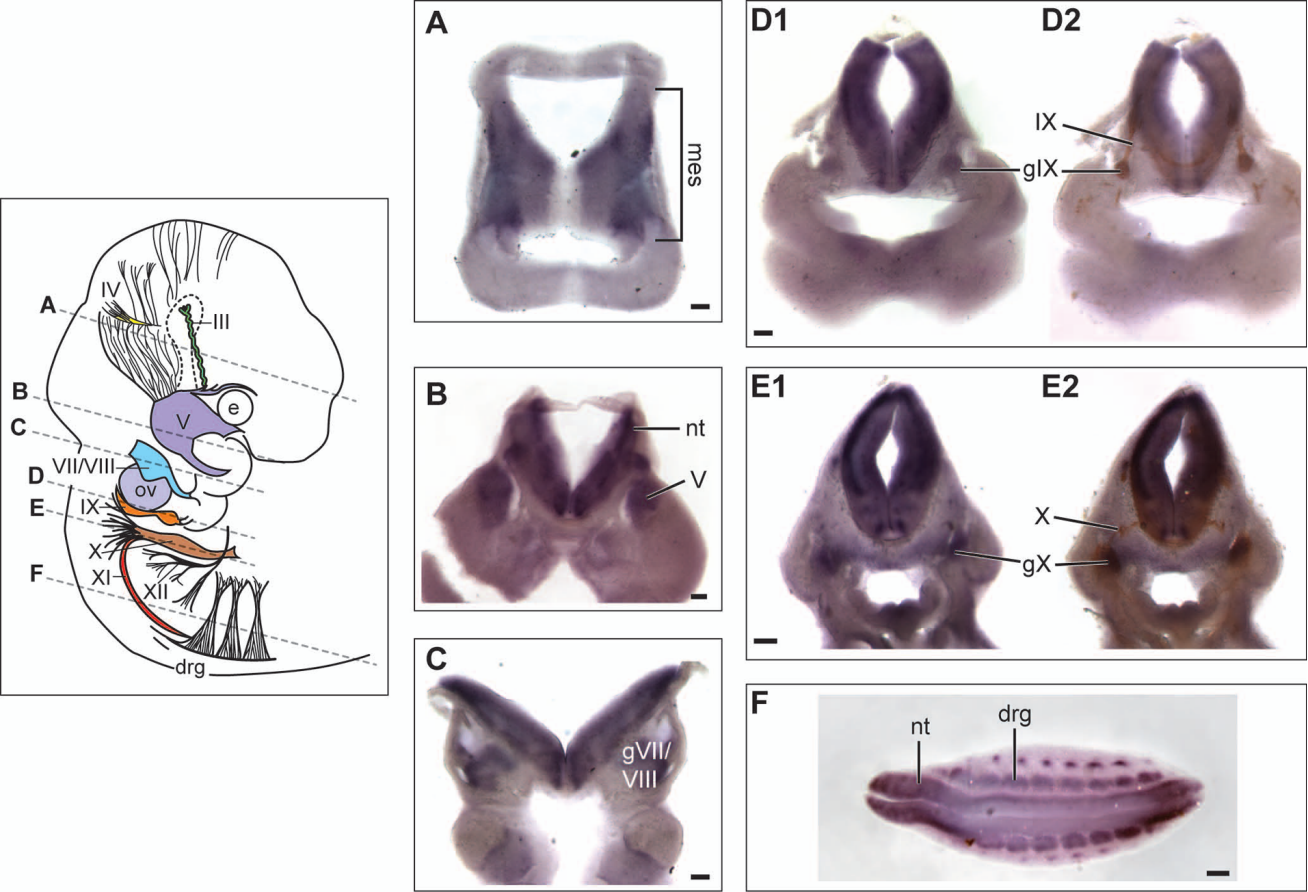
***Nav2 *is expressed in the mesencephalic tract, cranial and dorsal root ganglia and neural tube at E10.5**. **(A-F) ***Nav2 *mRNA expression in transverse vibratome sections of a wild-type embryo. Representative sections (200 μm) were analyzed by *in situ *hybridization using a riboprobe to *Nav2*. Staining for *Nav2 *is present in the mesencephalic tract (mes) neurons (A), trigeminal ganglia (V) (B), ganglia of VII and VIII (C), the ganglia of IX (D1) and X (E1) as well as the dorsal root ganglia (drg) (F). β-III tubulin antibody staining in vibratome sections (D2, E2) overlaps with that of the *Nav2 *riboprobe in the ganglia of cranial nerve IX (D1) and X (E1). The approximate location of the vibratome sections with respect to the whole embryo is shown by a dotted line in the illustration. e, eye; nt, neural tube; ov, otic vesicle. Scale bar: 200 μm.

The cranial nerve abnormalities observed here could arise from perturbation of several different developmental events, including abnormalities of early hindbrain patterning, neural crest cell migration, neuronal survival and/or elongation. *Nav2 *mRNA expression was found very early in the normal mouse embryo at the late-bud to early headfold stage and was similar to the distribution pattern described previously in the rat embryo (data not shown) [27]. At E10.5, robust expression of the *Nav2 *transcript was observed in the mesencephalic tract region (Figure 5A) as well as the ganglia of the trigeminal (V, Figure 5B), facial and vestibulocochlear (VII/VIII, Figure 5C), glossopharyngeal (IX, Figure 5D1) and vagus (X, Figure 5E1) nerves. Expression was also seen in the developing neural tube (Figure 5B-F) and the dorsal root ganglia (Figure 5F). At E10.5, a time point when neurites are extending from the ganglia, *Nav2 *expression co-localized with differentiating neurons identified by β-III tubulin staining in both the glossopharyngeal and the vagus ganglia (Figure 5D2 and 5E2, respectively). At earlier stages, *Nav2 *was found most abundantly in the neural tube and mesencephalon but was not detected in the region of the placodes or migrating neural crest (data not shown). Thus, the expression pattern of *Nav2 *was most consistent with either a role in hindbrain patterning and/or neuronal maintenance or elongation.

**Figure 6:**
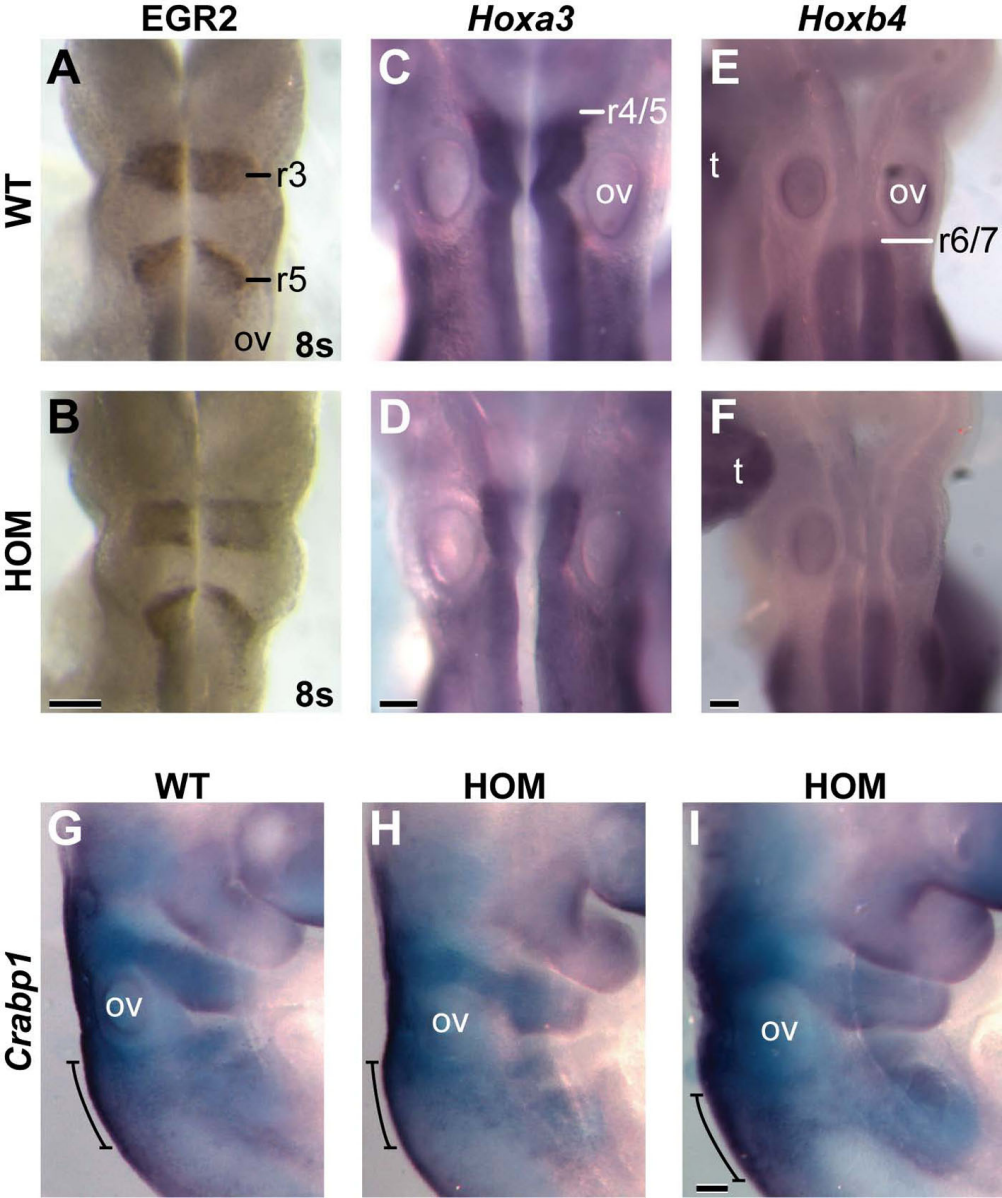
**Expression of EGR2 (KROX20), *Hoxa3*, *Hoxb4*, and *Crabp1 *is unaltered in the hindbrain of *Nav2 *homozygous mutant embryos**. Whole-mount immunohistochemistry for EGR2 shows staining in presumptive rhombomeres r3 and r5 at 8s in both **(A) **wild-type (WT) and **(B) ***Nav2*^-/- ^(HOM) embryos. **(C,D) ***In situ *hybridization analysis of *Hoxa3 *mRNA at E9.5 shows staining in the neuroepithelium caudal to the r4/5 border in both genotypes. *Hoxb4 *mRNA at E9.5 is expressed caudal to the r6/7 border in both **(E) **wild-type and **(F) ***Nav2*^-/- ^embryos. **(G-I) ***Crabp1 *mRNA at E9.5 is expressed in the neural crest cells, including those contributing to the IXth and X cranial nerves (bracket) in both wild-type (G) and *Nav2*^-/- ^(H-I) embryos. A total of ten 8s wild-type and *Nav2*^-/- ^embryos each were studied for EGR2 staining; 8 and 19, and 7 and 12 wild-type and *Nav2*^-/- ^embryos were examined for *Hoxa3 *and *Hoxb4*, respectively. Seven wild-type and 13 *Nav2*^-/- ^embryos were studied for *Crabp1 *staining. ov, otic vesicle; t, tail. Scale bar: 200 μm.

The hindbrain develops seven to eight transient lineage-restricted compartments called rhombomeres, and this segmented structure is closely involved in the development of the cranial nerves [40]. In order to determine whether an alteration in early hindbrain development might contribute to the CN defects observed in *Nav2*^-/- ^hypomorphic embryos, the expression of KROX20 (EGR2) protein, and *Hoxa3*, *Hoxb3*, and *Hoxb4 *mRNAs were studied. These transcription factors all play important roles in hindbrain patterning and also serve as markers of the presumptive rhombomere borders at early times in development. EGR2 is expressed in r3 and r5, and is required for the development of both of these segments [41]. *Hoxa3 *and *Hoxb3 *are expressed in the neuroepithelium caudal to the r4/5 border, and the disruption of *Hoxa3 *has been shown to cause defects in CN IX and X that are highly similar to those described here for the *Nav2*^-/- ^embryos [42]. *Hoxb4 *is expressed caudal to r6/7, serving as a useful marker for caudal hindbrain development [40]. When the expression of EGR2 was examined in embryos at 8s, similar immunostaining in r3 and r5 was observed both in *Nav2*^+/+ ^and *Nav2*^-/- ^embryos (Figure 6A, B). No difference in the staining pattern of *Hoxa3*, *Hoxb3*, and *Hoxb4 *was detected between wild type and homozygote mutants (Figure 6C-F and data not shown). These results indicate that hindbrain rhombomeric specification was not altered in homozygous *Nav2*^-/- ^hypomorphic mutant embryos. Neural crest cell migration from the hindbrain was examined by *in situ *hybridization using a probe to *Crabp1*, which is expressed in neural crest cells contributing to the IXth and Xth cranial nerves [43]. *Crapb1 *expression appeared similar in both homozygote mutants and wild-type embryos at E9.5 (Figure 6G-I), suggesting that neural crest cell migration defects may not account for the cranial nerve defects observed in the homozygous *Nav2 *mutant mice. These findings along with the expression pattern of *Nav2 *suggest that *Nav2 *may be exerting its effects on some aspect of neuronal elongation or survival rather than patterning or neural crest cell migration.

### The baroreceptor response is blunted in *Nav2*^-/- ^hypomorphic mutants

**Figure 7:**
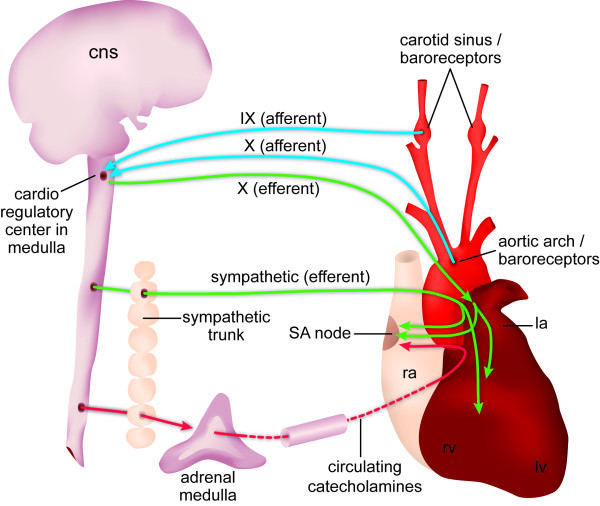
**Diagram of sympathetic and parasympathetic regulation of the baroreceptor reflex**. Schematic shows the nerves involved and areas innervated in the baroreceptor reflex arc. Adapted from [63]. CNS, central nervous system; la, left atrium; lv, left ventricle; ra, right atrium; rv, right ventricle; SA, sinuatrial.

Because both CN IX and X are involved in the regulation of cardiac function, and because these nerves were affected in the mutants, the baroreceptor reflex was compared in *Nav2*^-/- ^and *Nav2*^+/+ ^adult mice. The baroreceptor reflex is a mechanism to decrease heart rate when blood pressure rises and this reflex is dependent upon the function of both CN IX and X. Baroreceptors are stretch-sensitive mechanoreceptors; they are present in the carotid sinus, where they are innervated by CN IX, and in the aortic arch, where innervation by CN X takes place. When blood pressure rises, carotid and aortic distension occurs, leading to baroreceptor activation and signaling of the afferent fibers of CN IX and X to the brainstem. This leads to inhibition of the sympathetic branch of the autonomic nervous system as well as an increase in vagal (CN X) efferent signaling via muscarinic receptor activation to the heart. Thus, the heart rate is reduced to lower the blood pressure back to the baseline level. A decline in blood pressure operates in the opposite compensatory manner to increase heart rate to restore normal blood pressure. Figure 7 shows a schematic of the afferent and efferent nerve connections involved in the baroreceptor reflex.

**Figure 8:**
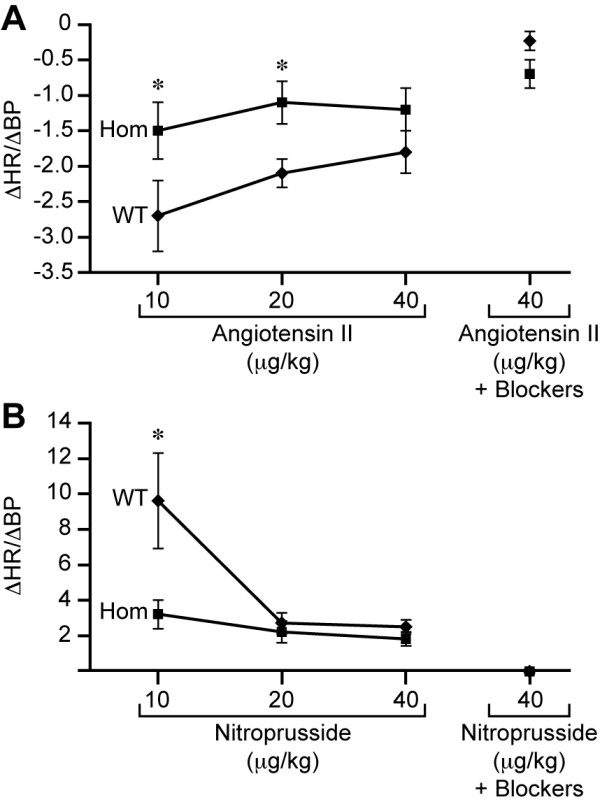
**The baroreceptor reflex response in response to angiotensin II and nitroprusside**. *Nav2 *hypomorphic mutants (Hom) show blunted responsivity (change in heart rate (ΔHR)/change in blood pressure (ΔBP)) at varying doses of **(A) **angiotensin II and **(B) **nitroprusside. Asterisks indicate statistical significance at *P *< 0.001 as determined by a two tailed *t*-test. WT, wild-type.

In order to test *Nav2*^-/- ^mice for this baroreceptor response, blood pressure changes were induced by pharmacological means using the vasoconstrictor angiotensin II (alpha 1 adrenergic agonist) and the vasodilator nitroprusside. The ability of *Nav2*^-/- ^mice to compensate for the drug-induced increase or decrease in blood pressure by decreasing and increasing heart rate, respectively, was compared to their wild-type counterparts. Initially, baseline data were recorded for at least 15 to 30 minutes, and these results showed there was no difference in systolic blood pressure or heart rate between the *Nav2 *hypomorphic mutants and wild-type mice prior to any drug intervention (Table 3). Mice were then treated with angiotensin II to induce an increase in blood pressure. In the *Nav2 *mutants, the expected decrease in heart rate in response to the change in blood pressure was significantly less than that observed in the wild-type mice at both the 10 μg/kg and 20 μg/kg doses of angiotension II (Figure 8A). Approximately 56% (5 out of 9) *Nav2*^-/- ^mutant mice showed a change in heart rate relative to the change in blood pressure (Delta value) below that of the lowest responder in the wild-type group in response to the 10 μg/kg dose of angiotensin II (data not shown). Thus, *Nav2*^-/- ^mice showed less ability to produce a compensatory reduction in heart rate in response to the vasoconstrictor effects of angiotensin II.

**Table 3:** Baseline heart rate and blood pressure in *Nav2 *hypomorphic mutant and wild-type mice

Genotype	Heart rate (beats/minute)	Systolic BP (mmHg)
Wild type	476 ± 21.3	92.9 ± 3.3
Homozygote	523 ± 21.5	90.4 ± 3.7

The baseline heart rate and systolic blood pressure (BP) are not significantly different when examining the pooled averages using a two-tailed *t*-test. Values are mean ± standard error of the mean.

Next, the vasodilator sodium nitroprusside was used to examine the responsiveness of *Nav2*^-/- ^hypomorphic mutant mice to a drop in blood pressure. Normally, a nitroprusside-induced drop in blood pressure should produce an increase in heart rate. However, a blunted heart rate response was observed in the hypomorphic *Nav2*^-/- ^mutants (Figure 8B). At the 10 μg/kg dose of nitroprusside, the hypomorphic mutant mice, on average, showed a heart rate increase only one-third that of the wild-type mice. At both the 20 and 40 μg/kg doses, wild-type mice became less responsive, and showed a change similar in magnitude to that of the *Nav2 *mutants. This is consistent with work showing the heart rate/blood pressure ratio normally declines with increasing doses of nitroprusside [44].

In addition to a reduced ability to increase or decrease heart rate appropriately in response to nitroprusside and angiotensin II, respectively, the *Nav2 *hypomorphic mutant mice showed a much longer duration of pressure change after both angiotensin and nitroprusside treatment that was most likely due to the blunted heart rate response (data not shown).

To determine whether the heart in *Nav2*^-/- ^mutants was able to respond appropriately to parasympathetic and sympathetic inputs, propranolol, a non-selective beta adrenergic receptor blocker (1 mg/kg) was used to block sympathetic contributions, and glycopyrrolate, a muscarinic receptor antagonist (100 μg/kg), was used to block parasympathetic input. Following propanolol administration, both wild-type and *Nav2*^-/- ^mice showed a reduction in heart rate consistent with blocking sympathetic input, and following glycopyrrolate, both groups showed an increase in heart rate consistent with blocking parasympathetic input. Thus, the responsiveness of *Nav2*^-/- ^mice to sympathetic and parasympathetic input from the medulla was intact. Furthermore, the magnitude of the blocker-induced changes in heart rate did not differ between the genotypes (Table 4). After application of the blockers, a 40 μg/kg dose of each of the drugs, angiotensin II or nitroprusside, was administered. There was a complete block of the baroreceptor response under both blocks with both treatment conditions, showing that adrenergic and muscarinic receptor response was blocked. Taken together, this work indicates that a defect in receptor signaling was not responsible for the diminished response in the *Nav2*^-/- ^mice. Overall, these baroreceptor studies showed that the hypomorphic *Nav2*^-/- ^mutant mice exhibited a blunted response to changes in blood pressure consistent with abnormal functioning of CN IX and X.

**Table 4:** Heart rate changes after sequential administration of a muscarinic receptor and a beta-adrenergic receptor blocker

	Baseline	Glycopyrrolate	Δ	Propranolol	Δ
Wild type	489 ± 22	526 ± 15	37 ± 16	448 ± 10	78 ± 15
Homozygote	522 ± 17	563 ± 17	41 ± 13	453 ± 19	110 ± 7

There is no significant difference between homozygous mutant and wild-type heart rate changes with either glycopyrrolate or propranolol. Values are mean ± standard error of the mean.

## Discussion

In the present study, nervous system development was studied in the *unc-53H2 *(*Nav2*) hypomorphic mutant mouse [32]. The results show that *Nav2 *is important for the normal development of neuronal fibers during embryonic development, and that the full-length protein plays a role in the development of CN IX (glossopharyngeal) and CN X (vagus). Postnatal survival is reduced in *Nav2 *hypomorphic mutant mice, and those that do survive to adulthood show a blunted ability to compensate for changes in blood pressure, a response requiring signaling through CN IX and X.

The observation that *Nav2 *hypomorphic mutant embryos show a reduction in overall nerve fiber density is consistent with the proposed role for this gene in neurite outgrowth [31]. In addition, it may help to explain how elimination of the full-length NAV2 protein contributes to the behavioral phenotypes reported previously in the *unc-53H2 *hypomorphic mutant mouse, including impaired acuity of several sensory systems. In adult mice, Peeters *et al*. [32] reported there was hypoplasia of the optic nerve; however, the morphology of the developing embryonic nervous system was not examined. In the present study, analysis of neurofilament antibody staining in *Nav2*^-/- ^(*unc-53H2*) embryos at E11.5, as well as analysis of nerve development at E13.5 in *Nav2*^-/-^/*Brn3a-lacZ*^+/- ^embryos, indicates that overall neuronal density, including that of the sensory neurons, is diminished. This was particularly evident in the trigeminal mesencephalic tract neurons and in the dorsal root ganglia. The dorsal root ganglia contain cell bodies of sensory neurons that are located along the spinal nerves and their processes are involved in sensing touch, stretch, temperature and pain. A reduction in the density of sensory neuron fibers in the *Nav2*^-/- ^mutants is consistent with previous work showing that these mice have impaired sensory function [32].

Consistent with the present observation of a reduction in nerve fiber density in *Nav2 *hypomorphic mutants, is strong evidence, both from work in cell culture and in *C. elegans*, supporting a role for *Nav2 *in neurite outgrowth and axonal elongation. In human NB cells, knock-down of full-length *Nav2 *eliminates atRA-induced neurite outgrowth, and ectopic expression of human *Nav2 *in the mechanosensory neuron rescues the ability of the *unc-53 *mutant to extend axons [31]. *Nav2 *has been proposed to function in neurite outgrowth by altering the structure of the cytoskeleton. The 261 kDa NAV2 protein contains several functional domains, including a calponin-homology domain at its amino terminus, several coiled-coil regions, a SH3-binding motif, a putative cytoskeletal-interacting domain, and a carboxy-terminal AAA-domain. In human NB cells, NAV2 is found closely associated with cytoskeletal elements, including microtubules and neurofilament proteins, and the cytoskeletal-interacting domain is required for NAV2 to interact with microtubules [31]. It has also been proposed that the neuron navigator family is involved in reorganizing the cytoskeleton to guide cell shape changes by serving as microtubule plus-end tracking proteins [45,46]. Based on work in *C. elegans*, UNC53 has been proposed to act as a scaffold that links ABL-1 to the ARP2/3 complex to regulate actin cytoskeleton remodeling [47]. Thus, although the molecular details concerning how *Nav2 *functions in neuronal elongation remain to be elucidated, the present study shows that *Nav2 *is essential for maintaining the density of neuronal fibers in the mouse, and that this function is exerted early in the developing nervous system of embryos.

In addition to nerve fiber density changes, the development of CN IX (glossopharyngeal) and CN X (vagus) are particularly affected in the *Nav2 *hypomorphic mutants. Nearly 60% of these mutants show a reduction in or lack of axonal connection between the distal ganglia of CN IX with the proximal ganglion and hindbrain, and/or a fusion of the distal ganglia of CN IX and X. The cranial nerves develop in register with lineage-restricted compartments called rhombomeres. Hindbrain development is a highly conserved and tightly regulated process in vertebrate animals and plays an important role in directing the pathways of neural crest migration ultimately producing cranial nerves and craniofacial structures that are integral to hindbrain function [48]. The disruption of a number of genes involved in the establishment and maintenance of rhombomeres are known to affect cranial nerve development [40]. However, analysis of selected markers of hindbrain segmentation did not reveal abnormalities in rhombomere identity in *Nav2 *mutants; thus, the defects in CN IX and X are not likely the result of defects intrinsic to the hindbrain neuroepithelium.

CN IX and X defects similar to those observed here in *Nav2 *hypomorphic mice have been reported in other genetic mutants, including *Hoxa3*, *CoupTF1 *, and *Sall3 *[42,43,49,50]. In a number of mutants showing fusion of CN IX and X, specification and segmentation of rhombomeres is reportedly normal, whereas perturbation of later developmental events involving precursor cell migration and/or axon extension have been proposed as a causative factor [49-51]. The cells that contribute to CN IX and X arise from the hindbrain (motor components), neural crest cells (NCCs) and the epibranchial placodes. NCCs arise from the dorsal edge of the neural tube and form both the proximal ganglion sensory neurons of CN IX and X, as well as the glial cells within the proximal and distal ganglia. The distal ganglion sensory neurons of CN IX and X are derived from epibranchial placodes. In addition to sensory nerves, both CN IX and X contain branchial and visceral (parasympathetic) motor components, with cell bodies in the ventral neural tube that project axons forming a bundle between the hindbrain and the proximal and distal ganglia. A change in migration of NCCs as assessed by *Crabp1 *expression at the level of r6/7 was not detected in the *Nav2 *mutants, leaving abnormalities in the dorsal ventral migration of placode-derived neuronal precursors, improper guidance of motor neurons from CN IX to CN X, and abnormalities in axonal guidance molecules as other possible factors that could contribute to the CN IX/X defects observed in *Nav2*^-/- ^embryos. In the present study, we have shown that the long *Nav2 *transcript (which encodes for the full-length protein) is expressed on or before E10.5 in the ganglia of CN IX and X, a time when NCC emigration from the neural tube and placode-derived cell migration is largely complete and when axon elongation is actively underway. The appearance of *Nav2 *in the forming ganglia of CN IX and X together with earlier work supporting the importance of *Nav2 *in neurite outgrowth supports the proposal that defects in cranial nerve development result, at least in part, from defects in axonal elongation/directionality, although a defect in precursor cell migration cannot be ruled out at this time.

The development of CN IX and X is also perturbed when retinoid signaling is disrupted [10,52]. Fusion of CN IX and X has been reported in *Raldh2 *null mutant mice given a restricted period of supplemental atRA from E7.5 to E9.5 [10], as well as in *Rar *α/β [52] compound null mutant mice. atRA plays an important role in hindbrain patterning at early stages of development, and the RAR α/β compound null mutant mice show profound alterations in post-otic rhombomere identities that most likely explain the CN changes. In contrast, atRA-rescued *Raldh2 *null mutant mice show no apparent defect in hindbrain patterning, indicating that the CN abnormalities must have resulted from the effect of retinoid deficiency on later developmental events [10]. atRA plays a role in the stimulation of neurite outgrowth [6] and defects in this process are seen in retinoid-deficient embryos. It is known that expression of *Nav2 *mRNA is regulated by atRA, both in cultured cells and in developing embryos [27]. Because *Nav2 *is required for atRA-induced neurite outgrowth [31] and is found in CN IX and X in early embryos, it seems plausible that *Nav2 *may lie downstream of atRA and its receptors in the regulation of normal CN development.

CN IX and X are integral components of the homeostatic mechanism called the baroreceptor reflex that is important in maintaining blood pressure. The carotid sinus baroreceptors are innervated by the distal (petrosal) component of the glossopharyngeal nerve (CN IX) and the aorta by the distal (nodose) component of the vagus nerve (CN X). The visceromotor or parasympathetic component of the vagus nerve (CN X) also plays a role in the baroreceptor reflex to slow heart rate. In a normal animal, there is a linear relationship between the stretch of the baroreceptor containing vessel wall and the afferent nerve discharges [53]. With a rise in blood pressure, the carotid and aortic sinuses become distended, the baroreceptors fire action potentials, and this activity travels back to the nucleus of the solitary tract in the brainstrem via the afferent (sensory) components of the glossopharyngeal and vagus nerves. This causes inhibition of the sympathetic branch of the autonomic nervous system, and excites the nucleus ambiguous (vagal nuclei) that regulate the parasympathetic nervous system, leading to the release of acetylcholine at the sinoatrial node, slowing heart rate and conduction. Conversely, a decrease in blood pressure decreases baroreceptor firing, leading to an increase in sympathetic outflow and decreased parasympathetic (vagal) outflow. The present work shows that the ability of *Nav2 *mutants to respond to either an increase or decrease in blood pressure is blunted. Mice treated with a vasoconstrictor (angiotensin II) do not show the same reduction in heart rate as their wild-type counterparts, suggesting that the afferents of CN IX and X that relay this information to the brainstem, and/or the vagal ouput needed to slow the heart is defective. When a vasodialator (nitroprusside) was given, the *Nav2 *mutants showed less ability to increase heart rate, again, consistent with a defect in the afferent component of CN IX and/or X. The defects observed early in *Nav2 *hypomorphic mutant embryos support the conclusion that the functioning of CN IX and X is altered in adult mice. However, because the baroreceptor reflex is a complex process involving multiple components, other factors, including the responsiveness of the vessels, could also be involved.

## Conclusions

We have provided *in vivo *evidence that the atRA-responsive gene *Nav2 *plays an important role in shaping the development of the mammalian nervous system. Elimination of the full-length *Nav2 *transcript and protein produces abnormalities in nerve fiber density as well as in the development of CN IX and X in early embryos. These CN defects may contribute to the reduced ability of adult *Nav2 *hypomorphic mutant mice to respond to acute changes in blood pressure due to abnormalities in the baroreceptor reflex.

## Methods

### Embryo generation

Embryos were generated from (*Nav2*^+/- ^× *Nav2*^+/- ^) and female *Nav2*^*+/- *^× male *Nav2*^*-/- *^crosses using C57BL/6-Tyrc-Brd un53H2 mice as described [32] and were further backcrossed into C57/BL/6 for up to six generations. *Brn3a *reporter mice were generated by crossing a *Brn3a-lacZ*^+/- ^mouse [38] with a *Nav2*^-/- ^mouse to generate *Nav2*^+/- ^/*Brn3a-lacz*^+/- ^mice; these were then bred to generate embryos for analysis that were *Brn3a-lacz*^+/- ^and *Nav2*^+/+^, *Nav2*^+/- ^and *Nav2*^-/-^. Noon the day of vaginal plug detection was considered E0.5. At early developmental times, embryonic stage was further defined based on somitic development as follows: 1 to 3 somites (E8.0), 4 to 6 somites (E8.25), 7 to 10 somites (E8.5) and 11 to 14 somites (E9.0). Embryos were obtained by cesarean section and genotypes determined by taking a sample of yolk sac (<E9.0), tail or limb (E9.5 and up). Samples were processed using the Direct PCR Lysis Reagent for mouse yolk sac or tail (Viagen Biotech, Los Angeles, CA, USA) per the manufacturer's directions. *Nav2 *genotyping was performed as described previously [32] and *Brn3a *genotyping was done as described in [39]. The *Brn3a *reporter mouse was a kind gift from Dr Eric Turner (La Jolla, CA, USA).

### X-gal staining of embryos

E12.5 and E13.5 embryos were fixed at room temperature in 4% paraformaldehyde (PFA) in phosphate-buffered saline (PBS) for 30 minutes and E15.5 embryos were fixed for 45 minutes. Embryos were then washed twice in PBS (pH 7.2 for all steps), and rinse buffer (5 mM EGTA, 2 mM MgCl_2_, 0.01% sodium deoxycholate, and 0.02% NP-40 in PBS), each for 15 minutes at room temperature; embryos were then placed in staining solution (5 mM K_3_Fe(CN)_6_, 5 mM K_4_Fe(CN)_6_, 5 mM EGTA, 2 mM MgCl_2_, 0.01% sodium deoxycholate, 0.5 mg/ml X-gal, 0.02% NP-40 in PBS). The embryos and tissues were incubated in the dark at 37°C for approximately 4 h or until the desired level of staining was reached. Embryos and tissues were washed in PBS for 10 minutes at room temperature, post-fixed in 4% PFA overnight at 4°C, washed again in PBS at room temperature for 10 minutes and stored in PBS at 4°C until they were photographed using a Nikon model SMZ-U dissection microscope fitted with a 1 × lens Qimaging camera and MetaMorph software (Molecular Devices, Downington, PA, USA).

### Whole-mount and vibratome *in situ *hybridization

A partial *Nav2 *rat probe was used for *in situ *hybridization studies as described in [27]; the riboprobe was 93% identical in nucleotide sequence to the mouse transcript (1,507-1,983 bp; NCBI accession number NM_001111016). The *HoxA3 *and *Hoxb3 *probes were a kind gift from Dr N Manley (Department of Genetics, University of Georgia, Athens, GA, USA), the *Hoxb4 *probe was generously provided by Dr R Krumlauf (Stowers Institute, Kansas City, MO, USA) and the *Crabp1 *probe was generated as described [54]. Whole-mount *in situ *hybridization was carried out using embryos fixed in 4% PFA by the methods previously described [55,56] with the following modifications. The proteinase K (pK)/refix times used at each stage were as follows: E8.5 (15/10 minutes), E9.5 (15/10 minutes; 10/8 minutes for *Crabp1*), E10.5 (20/15 minutes) for embryos in whole mount, and at 10.5 (8/10 minutes) for vibratome sections (200 μm). Both antisense and sense probes were tested; no specific staining with sense probes was observed (data not shown). *In situ *hybridization of floating vibratome sections (200 μm) was performed as described above except all incubations were performed in 24-well tissue culture plates. Sections were developed for 4 to 7 h on a rocker at room temperature. Embryos were processed and sectioned using a vibratome as previously described [57].

### Whole-mount immunohistochemistry

Whole-mount immunohistochemistry for 2H3 was performed as described [58,59] and for Krox20 (EGR2) using previously described methods [58,60]. Briefly, embryos (E10.5 for 2H3; 8s for EGR2) were fixed overnight in 4% PFA or Dent's fixative for 2H3 staining or in 4% PFA for EGR2. Embryos pretreated with H_2_O_2 _to quench endogenous peroxidase activity were incubated with either delipidated ascites, which were generated using a hybridoma (2H3) that secretes antibodies to neurofilament (Developmental Studies Hybridoma Bank, University of Iowa, Department of Biological Sciences, Iowa City, IA, USA) diluted 1:500 and 1:1,000 (for 4% PFA or Dent's fixed tissues, respectively) in TBST (10 mM Tris, pH 8.0, 150 mM NaCl, 0.05% Tween 20) or Krox20 antibody (Covance, Princeton, NJ, USA) diluted 1:1,000. This was followed by incubation with anti-mouse or anti-rabbit IgG conjugated to horseradish peroxidase (Southern Biotechnology Associates, Birmingham, AL, USA) diluted 1:1,000 in TBST. Embryos were pre-incubated in a solution of PBT containing 0.6 mg/ml 3,3'-diaminobenzidine (DAB; Sigma, St Louis, MO, USA) with 0.75% NiCl_2 _in PBT (PBS containing 0.05% Tween20) followed by the addition of H_2_O_2 _to a final concentration of 0.03% for color development. 2H3-stained embryos in BABB (benzyl alcohol:benzyl benzoate) were first scored whole (to ensure that basic information was collected in the event that subsequent handling destroyed the sample) and were then sliced along the sagittal midplane, rescored and photographed as described above. EGR2-stained embryos were photographed in PBS.

### Baroreceptor testing

Eighteen mice at 7 to 10 months of age, 9 male *Nav2*^*+/+ *^and 9 male *Nav2*^*-/- *^animals, from *Nav2*^*+/- *^× *Nav2*^*+/- *^crosses (backcrossed to C57BL/6 for four generations) were tested for baroreceptor function at the UCLA Mouse Physiology Laboratory.

For the baroreceptor reflex study, mice were anesthetized and then both femoral arteries were catheterized to obtain a continuous recording of pressure and to provide a port to infuse drugs using methods adapted from [61]. The electrical signals from the pressure transducer and EKG electrodes were connected to bridge amplifiers (AstroMed Grass Technologies, Warwick, RI, USA). The pressure and EKG signals were digitized and displayed with HEM V4.0 software (Notocord Systems, Croissy-sur-Seine, France). All pressures were calibrated with a Hg manometer (Baumaster, FL, USA). Heart rates were calculated during the data acquisition by the HEM program from the R-R intervals of the EKG and from the arterial pressure waves.

The baroreceptor responsivity was assessed by a sequence of small bolus infusions (0.08 to 0.12 ml) of angiotensin II and sodium nitroprusside. First angiotensin II was given in three increasing doses of 0.5, 1.0 and 4 μg/kg. Infusions were separated by at least 5 minutes to permit drug wash out and re-stabilization to control pressure and heart rate levels. This was followed by the administration of three doses of sodium nitroprusside administered at 10, 20, and 40 μg/kg. After at least 5 minutes of restabilization, propranolol was administered at 1 mg/kg. This was immediately followed by an infusion of glycopyrrolate at 100 μg/kg. After these autonomic blockers were administered and the animal stabilized, the high doses of angiotensin and sodium nitroprusside were repeated as controls. At the completion of the sequential infusions, the mouse was euthanized.

### Animals

All animals were maintained according to conditions under a research protocol approved by the Institutional Animal Care and Use Committee at the University of Wisconsin-Madison. The baroreceptor studies were performed according to conditions under a research protocol approved by the UCLA Chancellor's Animal Research Committee.

### Statistics

Statistical significance was determined by Chi-square analysis, Student's *t *test, Z score testing or two-way analysis of variance (ANOVA). Student's *t *test was done with Microsoft Excel, while *P*-values were generated from Z scores using P-Value Calculator [62]. Data are represented as averages of the mean ± standard error, unless noted otherwise.

## Abbreviations

atRA: all-*trans *retinoic acid; CN: cranial nerve; E: embryonic day; Nav2: neuron navigator 2; NB: neuroblastoma; NCC: neural crest cell; P: postnatal day; PFA: paraformaldehyde; PBS: phosphate-buffered saline; r: rhombomere.

## Competing interests

The authors declare that they have no competing interests.

## Authors' contributions

EMM participated in study design, carried out studies of embryos, interpreted experiments and wrote the manuscript. KPR conducted baroreceptor response testing and interpretation. DM provided the *Nav2 *hypomorphic mouse strain. MCD participated in study design, interpretation and critical evaluation and revision of the manuscript.
